# Recent Advances in Development of Functional Spider Silk-Based Hybrid Materials

**DOI:** 10.3389/fchem.2020.00554

**Published:** 2020-06-30

**Authors:** Aleksandra P. Kiseleva, Pavel V. Krivoshapkin, Elena F. Krivoshapkina

**Affiliations:** Laboratory of Solution Chemistry of Advanced Materials and Technologies, ITMO University, St. Petersburg, Russia

**Keywords:** spider silk, organic-inorganic hybrids, biomaterials, functional materials, silk protein materials, nanoparticles

## Abstract

Silkworm silk is mainly known as a luxurious textile. Spider silk is an alternative to silkworm silk fibers and has much more outstanding properties. Silk diversity ensures variation in its application in nature and industry. This review aims to provide a critical summary of up-to-date fabrication methods of spider silk-based organic-inorganic hybrid materials. This paper focuses on the relationship between the molecular structure of spider silk and its mechanical properties. Such knowledge is essential for understanding the innate properties of spider silk as it provides insight into the sophisticated assembly processes of silk proteins into the distinct polymers as a basis for novel products. In this context, we describe the development of spider silk-based hybrids using both natural and bioengineered spider silk proteins blended with inorganic nanoparticles. The following topics are also covered: the diversity of spider silk, its composition and architecture, the differences between silkworm silk and spider silk, and the biosynthesis of natural silk. Referencing biochemical data and processes, this paper outlines the existing challenges and future outcomes.

## Introduction

Nature is rife with nanocomposites, which exhibit high toughness and are found in various tissues—from abalone shells (Smith et al., 1999) to human bones (Ji and Gao, 2004). Spider silk, one of the most incredible natural hierarchically ordered materials, possesses outstanding material properties, namely high toughness (about three times higher than Kevlar toughness), high extensibility (30% elongation to fracture) equivalent to rubber elongation, and biocompatibility (Vollrath, 2000; Allmeling et al., 2006; Porter et al., 2013). Generally, spider silk is a highly ordered protein fiber spun by spiders. In-depth understanding of natural structuring and synthesis highlights the importance of hierarchical structures in terms of real world functionality (Buehler, 2013). This denotes thrilling prospects concerning the idea of converting the properties of hierarchically ordered structures to novel material functions. This approach generates opportunities for innovative material applications in the fields of energy and sustainability, medicine, and nanobiomedical technology (Zhang, 2003; Barthelat, 2007; Aizenberg, 2010; Hauser and Zhang, 2010; Salgado et al., 2010). It is therefore unsurprising that spider silk is considered one of the most promising materials for industrial applications. Silk is also attractive in optics and photonics (Huby et al., 2013), and tissue regeneration (Bandyopadhyay et al., 2019). Additionally, the mild conditions of its biosynthesis imply that the fabrication of innovative functional silk-based smart materials would be an eco-friendly process with minimal negative ecological impact.

Within the last few years, there has been a dramatic increase in the use of natural fibers to create new hybrid materials (Lau and Cheung, 2017). Recent advances in natural fiber development, composite science, and genetic engineering have presented remarkable opportunities for novel high-performance functional materials (Wang F. et al., 2014). There is a growing interest in high-performance spider silk-based functional materials, while silkworm silk is widely accepted and used. In this context, the paper summarizes recent progress in spider silk-based organic-inorganic hybrid material synthesis. To provide a more comprehensive understanding of spider silk, the structure–property–function relationships of spider silk fibers are discussed. Considering recent findings, the transformation of silk proteins into highly efficient fibers is also explored. Information regarding spider silk architecture is also extended to advance the targeted design of ultra-performance functional materials based on fibrous proteins.

This paper focuses solely on spider silk-based hybrids. Therefore, the structure and properties of silkworm silk, as well as those hybrids based on it are not the subject of this review. Differences between structure and material performance regarding silkworm and spider silk are explored, however, to provide a deeper understanding of silk backbone performance in the fabrication of silk-based functional materials. Namely, their organization at the molecular level, interactions to form secondary structures, and various mechanical properties are highlighted. Challenges and approaches to the large-scale production of spider silk-based materials for numerous applications are also reviewed. Additionally, the high complexity of spider silk organization, and the tunability of its properties are revealed. Recent advances and emerging strategies concerning the fabrication and applications of natural or bioengineered spider silk–inorganic nanoparticle hybrid materials are described. Lastly, this paper concludes with the prospects of hybrid spider silk-based materials.

## Composition–Structure–Property–Function Relationship of Natural Spider Silk

**Figure 1:**
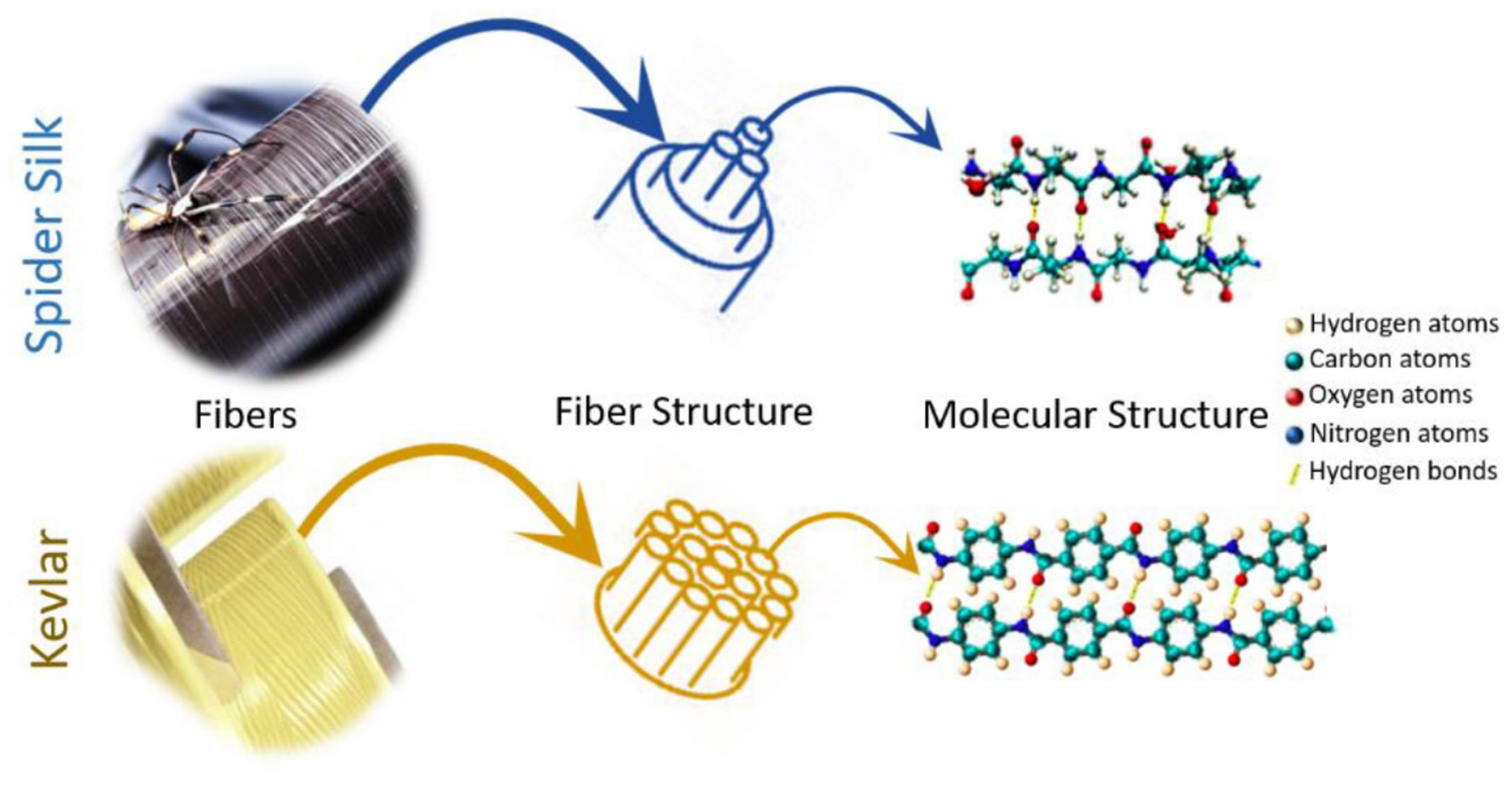
Comparison between spider silk and Kevlar fibers.

The marked interest of material scientists in the natural silk produced by spiders can be attributed to the broad spectrum of its extraordinary mechanical properties (high fracture strength, high stiffness, exceptional extensibility, and toughness) (Shao and Vollrath, 2002; Du et al., 2011), slow biodegradability (Vasconcelos et al., 2008), and high biocompatibility (Hakimi et al., 2007; Vepari and Kaplan, 2007). Despite high-performance synthetic silk analogs, such as nylon or Kevlar, being chemically similar to it (i.e., polyamide containing highly repetitive structure), the toughness of natural silk is outstanding (up to 180 MJ/m^3^) and far superior to any other biological or engineered material. Though Kevlar is a polyaramid fiber whereas spider silk is a polypeptide fiber, the molecular structures of Kevlar and spider silk are quite similar as seen in Figure 1. When spun into a fiber, Kevlar has a crystalline arrangement with its polymer chains oriented along the fiber axis. In its structure the amide groups keep separate polymer chains together forming hydrogen bonds between the polymer chains. These features are also typical of spider silk. Since spider silk is mostly used to catch fast flying insects, it is not surprising that it is much more elastic but weaker than Kevlar (Sirichaisit et al., 2000; Davies et al., 2013). Due to this, spider silk absorbs considerably more energy prior to breaking than any common synthetic material. In general, stress can be defined as a quantity that reflects the internal forces that neighboring material particles exert on each other, whereas strain signifies the degree of material deformation. Therefore, one of the most significant mechanical properties of a polymer fiber is its tensile stress–strain behavior. It shows fiber response to mechanical deformation along the fiber axis and is restrained by the capability of the molecules in the material to tolerate the applied stress, that is, when the fiber is stretched.

Noteworthy, numerous reports highlight the different performances of various types of silk fibers (Doblhofer et al., 2015; Andersson et al., 2016). Due to its superior mechanical properties, spider silk tends to be stronger and tougher than silkworm (*Bombyx mori*) silk, though both silks are composed of similar glycine-rich proteins (Hakimi et al., 2007). The issue of different silk performance has been the subject of much research. Most case studies agree that it is the different chemical composition of the silks, distinct amino acid distribution, and fiber microstructures that determine silk material properties (Saravanan, 2006; Römer and Scheibel, 2008; Nova et al., 2010).

These ideas might be justified by the fact that the silk fibers of a single species serve different biological needs, and therefore demonstrate specific mechanical characteristics (Vierra et al., 2011). The structural diversity of spider silk, in accordance with its various biological purposes, is one of their most outstanding characteristics. For instance, seven distinct silk-producing glands, each responsible for producing their unique gland-specific silk proteins for particular fiber types with diverse functional properties, were identified in orb- or cob-weaving spiders alone (Guerette et al., 1996). The major and minor ampullate glands produce dragline and structural silk for auxiliary spider web formation. Pyriform glands are responsible for producing silk fiber for attachments, while tubuliform glands provide female spiders with spider silk for making egg sacs. Flagelliform glands are very important since they allow the production of silk used as the main component of the capture spiral. Aciniform glands are used to form silk fibers for the wrapping and immobilization of prey, for web decorations and for lining burrows. The product of aggregate glands is aqueous secretions that act as glue for prey capture (Tokareva et al., 2014).

The chemical composition, structure, properties, and functions of some common types of spider silk have been described to a variable degree (Lewis, 2006). For example, flageliform silk, also known as capture spiral silk, is over ten times more extensible than the dragline silk that serves as a lifeline for spiders in web frame construction and locomotion. When exposed to even an insufficient force, flageliform silk can extend up to 200–300% its original length. Meanwhile, flageliform silk cannot rival dragline silk in strength and stiffness (Blackledge et al., 2005). Although the majority of the studies on spider silk focuses only on spider dragline silk, biochemical analyses demonstrate that the outstanding mechanical properties of spider silk originate from their unique primary amino acid sequences, produced by expressing specific spider silk genes in certain silk-producing glands (Vierra et al., 2011). Furthermore, these amino acid sequences are arranged in domains, in which more crystalline and less crystalline polypeptides are mixed in different proportions for various silk types (Gosline et al., 1999; Römer and Scheibel, 2008).

As a result, it has become evident that the chemical structure itself determines spider silk properties. In this context, in the past two decades substantial research has been dedicated to understanding the relationship between the molecular structure and functional properties of silk (Yarger et al., 2018). These data enable a more holistic discussion of its structure–property–function relationships, thus providing more in-depth knowledge to advance the material design.

### Distinction Between Silkworm and Spider Silks

It is particularly important to highlight the key differences in silkworm and spider silk performance to justify the distinction rationale. Spider dragline silk fibers are generally stronger (demonstrating higher maximum stress before breaking) and more extendible (demonstrating higher strain) than those of *Bombix mori (B. mori)* silk (Andersson et al., 2016). Additionally, spider dragline silk fibers have a very high strength-to-density ratio, and can absorb great impact energy (Du et al., 2011), while *B. mori* fibers have weaker mechanical properties (Shao and Vollrath, 2002). This contrast in macroproperties is justified by the difference in the composition and microstructure of these silks.

In general, a strength advantage of spider dragline silk over *B. mori* silkworm silk is due to the specific composition of amino acid sequences that form highly repetitive domains of both fibroin (the main protein of silkworm silk) and spidroin (the main protein of spider silk) (Pérez-Rigueiro et al., 2000; Andersson et al., 2016). At the molecular level, silk proteins of different origins vary greatly in their amino acid sequence and their interactions to form secondary structures. Moreover, different types of secondary structures result in different mechanical properties (Lefèvre et al., 2007).

From a design point-of-view, silks of diverse origins are primarily large proteins (~250–350 kDa) that consist of two parts: a main highly repetitive hydrophobic core domain, and non-repetitive hydrophilic amino- and carboxy-terminal domains (N-and C-termini, respectively) (Vollrath et al., 1996; Rising et al., 2006). Notably, the composition of the core region is rich in amino acids, which is ideal for assembling β-strands to form crystalline β-sheet secondary structures.

It is important to stress the molecular backbone of silk proteins comprise a polyamide chain that, along with amines, includes amino acid residues, which differ in charge, size, chemical reactivity, and hydrogen bonding capacity. These amino acid residues, being specific for spidroins (mainly alanine (Ala), glycine (Gly), and glutamic acid) and fibroins [most frequently alanine (Ala), glycine (Gly), and serine(Ser)], are small in size. Moreover, they reduce the inter-chain distance and, consequently, provide higher density, resulting in improved mechanical properties (Colomban and Dinh, 2012).

Structural analysis of silk fibers confirms that β-sheet secondary structures constitute the backbone of its crystalline domains, which are highly interspersed between the amorphous regions formed by helical structures and other secondary structural elements of silk protein (Keten and Buehler, 2010). Both the mechanical strength and extensibility of silk proteins are determined by the total amount of both β-sheet nanocrystallites and non-crystalline amorphous regions within the protein, respectively (Hayashi et al., 1999; Keten and Buehler, 2010). Whereas, the β-sheet content in fibroin is 40–60%, its crystalline secondary structures are typically formed by repetitive Gly-Ala-Gly-Ala-Gly-Ala-Ser motifs. Meanwhile, in spidroins of *Nephila* dragline, β-sheets constitute only 36–37% of its total protein content, and are formed by long chains of poly(Ala) and poly(Gly-Ala) residues (Gosline et al., 1999; Lefèvre et al., 2007). *B. mori* silk fibers therefore have both a higher tensile strength and Young's modulus (due to high β-sheet content) than spider silk fibers, since Young's modulus values are related to crystalline structure content (Keten et al., 2010). From an architectural standpoint, however, the β-crystallite density of spider dragline silk is almost twice that of *B. mori* silk, making the β-sheet regularity of spider dragline silk better than that of *B. mori* silk. Due to the abovementioned structural crystal factors, spider silk fibers are much stronger than those of the *B. mori* silkworm (Lin and Liu, 2015).

Dragline silk spidroin β-sheets are more mechanically stable due to the greater content of alanine-rich repetitive domains (Simmons et al., 2010; Malay et al., 2016) that provides the favorable interlocking mechanism of the side-chain methyl groups of alanine relative to glycine (Regan, 1994). The reason for this is that, in contrast to the repetitive poly(Ala) motif, alanine residues are placed on alternating sides of the protein backbone. Due to this conformation, poly(Ala) chains are bonded via hydrophobic interactions with protruding methyl groups located in an empty space near the alpha-carbon on the neighboring chain. Consequently, the β-sheets have no empty space and are therefore impervious to water. In contrast, although poly(Gly-Ala) regions form a similar secondary structure, the glycine side chain is unable to produce the same hydrophobic interactions as the alanine side chain. Therefore, β-sheets primarily formed by poly(Gly-Ala) regions have fewer links, resulting in their lower density and different mechanical properties (Eisoldt et al., 2011).

To compensate for having fewer β-sheets, spidroin is counterbalanced by an additional 10–15% more flexible secondary structures not found in fibroins, such as β-turns, 3_1_-helices, and random conformations that contribute to the extension of silk fibers. At the same time, the 3_1_-helical structures of dragline spidroin consist of the repetitive Gly-Gly-X (with X representing Ala, Asp, Tyr) amino-acid motifs, which on the one hand supports the structural strengthening of the well-ordered β-sheet nanocrystallites while providing extensibility to the silk fibers on the other (Gosline et al., 1999). These molecular interactions explain spider silk superior strength over silkworm silk, making it one of the most durable natural materials. Compared to fibroin, spidroin contains larger portions of amorphous domains primarily composed of glycine-rich sequences, which account for the elastomeric properties of silk proteins (Rauscher et al., 2006). In contrast, silkworm silk shows considerably lower extensibility due to the low ratio of the amorphous domain (Lefèvre et al., 2007).

When compared to spidroin that shows ductile characteristics, silkworm fibroin exhibits characteristics consistent with relatively brittle materials due to the presence of serine amino acid residues (specific for silkworm fibroin) that affect its mechanical properties and material behavior mainly through the formation of more hydrogen bonds. Notably, during the fracture of silkworm fiber, hydrogen bonding in fibroin dramatically decreases, which, given the decisive role played by hydrogen bonds in the structural stability of silk proteins, results in said brittle material characteristics. (Lee et al., 2016).

Thus, the superior mechanical properties of spider silk to silkworm silk are due to the differences in composition and supramolecular organization of base units in silks (Vollrath and Knight, 2001; Pérez-Rigueiro et al., 2007). A number of studies suggest that, for the same type of material, the degree of crystallinity, water content, degradation and sample preparation directly correlate to the resulting material behavior (Du et al., 2006; Holland et al., 2006; Murugesh Babu, 2017). In this regard, it can be argued that the macroproperties and, therefore, the functions and behavior of these two silks differ greatly, providing various choices in the field of functional materials fabrication.

### The Strong Relationship Between Architecture and the Biophysicochemical Properties of Natural Spider Silk

In general, structure–property relationship is of great importance in polymer science. For biomaterials, scientists are particularly interested in the correlation between the behavior, properties, and corresponding structure of the material. Natural evolution has achieved excellent results through the self-assembly of hierarchical structures at the molecular, nano- and microscopic levels (Du et al., 2011; Fu et al., 2011; Xu et al., 2014; Nguyen et al., 2015). To advance the production and application of spider silk-based materials, it is necessary to enhance their tunability into highly efficient architectures extremely dependent on the relationships between structure and properties of silk proteins (Ling et al., 2018; Mehrotra et al., 2019). In this regard, substantial research has been devoted to understanding the correlation between the molecular and structural architecture of spider silk and its biophysical and chemical properties, with the intention of developing ultra high-performing, smart and multifunctional materials (Ling et al., 2018).

The architecture of the dragline spider silk fiber is still a subject of discussion. From an experimental perspective, the fiber has been assumed to exhibit two-layer “skin core structure” (Frische et al., 1998; Augsten et al., 2000; Andersson et al., 2013), four-layer (Vollrath et al., 1996) and also five-layer structures (a lipid coat, a glyco coat, a skin, an outer core, and inner core) (Sponner et al., 2007). The lack of convincing data on spider silk architecture is probably due to the fact that these studies involved extensive diverse silk processing, including dehydration, cyclic freeze-thawing, treatment with urea, esters, or Triton x-100 prior to structural analysis. Simultaneously, several structure–property models of spider silk were created using modeling methods based on the conventional polymer theory, including the two-phase crosslinking network model (Termonia, 1994), the order/disorder fraction model based on the middle field theory (Porter et al., 2005), and the Maxwell model (Krasnov et al., 2008). Nevertheless, a significant drawback of these models is their consideration of silk as a homogeneous polymer fiber while its physical characteristics correlate to the primary and/or secondary structures of silk amino acids.

However, most studies on silk fiber architecture have agreed on the presence of micro- and nanofibers along the fiber axis within the silk fiber, improving its tensile and strength properties (Li et al., 1994; Miller et al., 1999; Sponner et al., 2007). In support of this, relaxation studies on natural spider silk pointed toward torsional memory in silk, which may in turn indicate a high degree of heterogeneity in natural dragline silk fibers (Emile et al., 2006). Shortly afterwards it was found that a certain heterogeneity in fiber architecture improves fiber strength, whereas deviations in heterogeneity can lead to fibers being less durable (Cranford, 2013).

**Figure 2:**
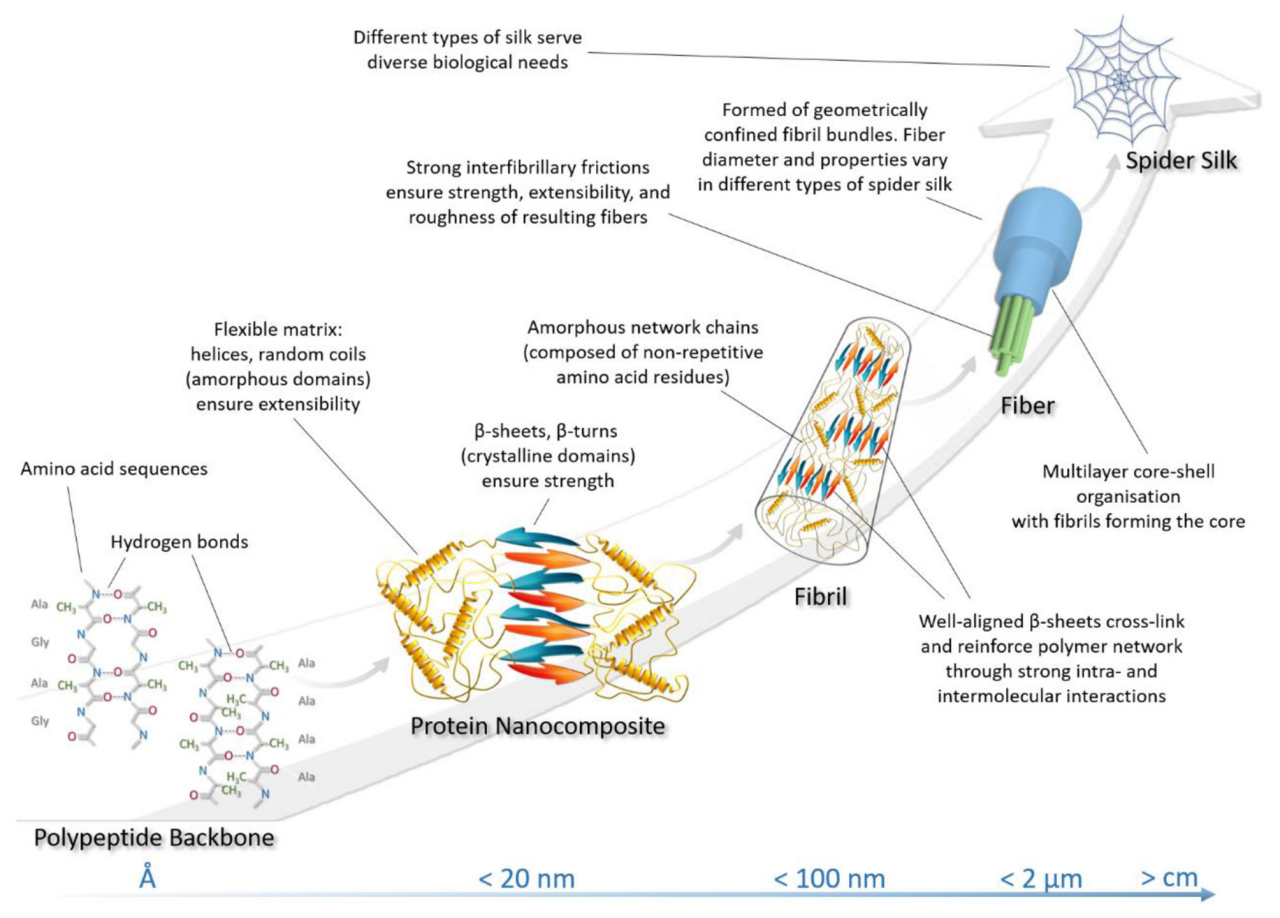
Structural hierarchy in spider silk architecture.

Significant evidence from both modeling and experimental studies have shown that natural spider silk achieves its outstanding mechanical properties through the hierarchical design of nanostructures (Figure 2) (Keten and Buehler, 2010; Giesa et al., 2011). Different types of spider silk show a common characteristic nanostructure (Yarger et al., 2018). Briefly, the nanostructure of the fiber is constructed from hierarchical nanofibrils with nanoscale defects between these fibers. (Frische et al., 1998; Lin and Liu, 2015). However, as mentioned above, the unique self-assembly of the secondary protein structures, the distribution of nanoscale defects, and the hydrogen bonding present in the crystalline and semi-amorphous domains determine the nanoscale behavior of silk protein assembly, and contribute various characteristic features to the resulting fiber (Keten and Buehler, 2010; Wang et al., 2018a).

In terms of spider silk fiber architecture, the lowest level of silk hierarchy is rooted in its primary protein structure and is defined by a sequence of amino acid residues responsible for the sequential folding mechanisms leading to its molecular structure (Keten and Buehler, 2010). The amino acid sequence of the primary protein structure dictates the chemistry of higher-order secondary protein structures. Furthermore, dispersed hydrodynamic low-density hydrogen bonds are responsible for assembling more amorphous helical and elastic domains, while the denser hydrogen bonding controls the characteristic size and strength of the crystalline β-structures (Tarakanova and Buehler, 2012). The effect of amino acid sequence on the appearance of hydrogen bonds was discussed in the previous section. Crystallites also make a major contribution to the strength of silk fibers (Wang and Schniepp, 2018). At a higher hierarchical level, these two regions establish a joint interaction in such a way that β-sheet nanocrystallites are embedded in an amorphous grid of less-ordered secondary protein structures. It is important to note that the β-crystallites in silk nanofibrils are the cross-linkers responsible for the extra toughness of silk filaments (Liu et al., 2016). Thus, the spatial arrangement of secondary structures forms a higher order protein structure, the so-called protein nanocomposite, which directly affects complementary functions (such as strength and extensibility) of the resulting fiber (Giesa et al., 2011). These nanocomposite structures in turn form nanofibrils, which are nanoscale molecular networks of nanocomposites together with molecular silk chains, geometrically bounded in diameter by 30–80 nm (Mehrotra et al., 2019). The orientation of nanocrystallites within these structures profoundly affects the breaking stress of silk fiber materials. Thus, it was found that the more ordered and well-aligned the β-crystallites within the structure of nanofibrils are, the more they enhance the fiber strength (Wang and Schniepp, 2018). Strong β-sheet–β-sheet interactions and high density of β-crystallites in nanofibrils also strengthen silk fibers (Liu et al., 2016). Bundles of twisted nanofibrils with strong interfibrillary friction bind together into the silk fibers that form spider silk threads. The nature of the cohesive interactions between nanofibrils dictates the strength, extensibility, and roughness of the final silk fibers (Giesa et al., 2011; Lin and Liu, 2015). Table 1 summarizes the structural hierarchy of spider silk.

**Table 1:** Summary of structural hierarchy of spider silk with structural roles and properties.

**Hierarchical level and structure**	**Structural role**	**Key properties and mechanisms**
(1) Primary protein backbone of amino acid sequences (Å scale)	The primary protein structure is composed of a sequence of amino acid residues that define the folding of subsequent hierarchical levels	Basic building block ensuring and dictating resulting biophysicochemical properties of the fiber
(2) Secondary protein structure of β-sheet nano-crystals are comprised of poly(Ala) and poly(Gly-Ala) sequence motifs (from 2 to 4 nm) (Du et al., 2006)	β-sheet crystals exhibit exceptional strength due to high density of hydrogen bonding between the sheets	Cooperative hydrogen bonds improve crystal toughness via self-healing capacity of hydrogen bonding providing the basis for larger-scale toughness and strength (Keten et al., 2010)
(3) Protein nanocomposite (β-sheets, β-turns, helices) (from 10 to 20 nm)	Nanocomposite structure is the three-dimensional arrangement of interacting secondary structures.	The interplay between β-sheet crystals and amorphous domains ensures strength and extensibility of the nanocomposite structure. The two domains establish a collaborative interaction contributing complementary function: strength and extensibility (Gosline et al., 1999; Keten and Buehler, 2010; Nova et al., 2010)
(4) Fibrils (about 100 nm long, 50 ± 30 nm in diameter)	The coherent interaction of the protein domains that make up the fibrils is responsible for uniform load distribution	The network-like structure within the fibril leads to uniform deformation of all protein domains within the fibril, enhancing strength and toughness due to the contribution of many protein components acting together
(5) Fibers 500 nm−3 μm in diameter (Du et al., 2006)	Bundles of fibrils connected into fibers increase the mechanical properties of silk threads	The binding of several co-directional fibrils into a single fiber increases the properties of the material by achieving a uniform deformation state (flaw tolerance) (Giesa et al., 2011).

Here, it has been brought to light why spider silk is a unique natural material and an outstanding hierarchical model for understanding how silk proteins are able to become highly efficient fibers. Understanding the hierarchical organization of spider silk fibers clearly justifies the directional design and synthesis of ultra-performance materials based on fibrous proteins in a more general way. Moreover, this comprehensive understanding of spider silk architecture provides the instruments necessary for efficiently determining material composition and properties at all levels to attain exceptional material performance in various industries. Better understanding of the structure-property relationship in silk allows new approaches to manipulating the material at all scales and the possibility of controlled construction of smart hierarchical protein materials with improved properties.

### Scalability of Spider Silk Production and Related Problems

A prerequisite for the widespread applications of spider silk is its mass production. In this regard, the challenges of large-scale spider silk-based material production are highlighted in this section. Despite the fact that silkworm silk has been used for millennia and its large-tonnage production is well-established, spider silk production is not as straightforward as it could be. From an industrial standpoint, there are several reasons why spider silk has not received the same wide attention as silk from silkworms. Primarily, this issue is directly related to the difficulties associated with establishing and maintaining large and dense spider populations due to their predatory and cannibalistic nature. Unlike silkworms, spiders are able to produce only small amounts of silk, which, furthermore, cannot be manufactured as a single fiber (due to the fact that while spinning, spiders use several types of silk as has been mentioned earlier).

In this regard, new biotechnological formulations of alternative approaches for producing spider silk are emerging (Kluge et al., 2008). Significant progress in genetic engineering aimed at creating materials with structures and properties similar to those occurring in nature has resulted in advanced technologies for producing synthetic spider silk fibers. However, the bioengineered production of recombinant spider silk on a commercially viable scale remains a challenge. Although some sequences of the spidroin genes have been researched (Babb et al., 2017), the full implementation of recombinant technology (the transfer of silk genes to bacterial or yeast hosts for protein expression) for spidroin production still presents certain challenges. When using recombinant technology, replication of the full length of recombinant spider silk proteins is oftentimes complicated, since the large length and the size of various spidroins hinder their synthesis and secretion by bacterial hosts (Teulé et al., 2009; Lin et al., 2013; Tokareva et al., 2013; Doblhofer et al., 2015; Rising and Johansson, 2015). The subsequent isolation and purification of spidroins is also impeded (due to the extremely low solubility compared to native spidroins) (Xu et al., 2012; Copeland et al., 2015; Zhang et al., 2015). Consequently, recombinant production often results in the fabrication of significantly shorter proteins with lower molecular weights that, in many cases, contain only a small portion of the repetitive region and lack one or both terminal domains (Rammensee et al., 2008; Heidebrecht and Scheibel, 2013). Such replicas behave much differently from natural spider silk proteins (Eisoldt et al., 2011).

In parallel, polymer engineering technology has also been utilized on a commercially viable scale in an attempt to replicate the properties and structure of natural spider silk fibers (Andersson et al., 2017; Blamires et al., 2017). Mainly, biomimetic approaches have been proposed for the transition to large-scale silk fiber production, providing some parallels between the production of synthetic polymer fibers and the natural fiber spinning. Some reports describe efforts to imitate the formation of natural spider silk fiber by pumping highly viscous liquid polymers through microscopic spinnerets at a flow rate that mimics that of the natural process (Kojić et al., 2006; Holland and Vollrath, 2008). Generally, such approaches rely on the extrusion process during artificial fiber spinning to mimic the molecular orientation and bonding observed in natural silk fibers (Lefèvre and Auger, 2016). Due to difficulties in simulating all the natural physiological and biochemical spinning processes in the laboratory, perfect replication of the natural spinning process is not currently possible (Blamires et al., 2012). These recent reviews expound on the subtleties and complexities of the bioengineering and biomimetics of spider silk (Andersson et al., 2016; Blamires et al., 2020).

As is reflected in the poor mechanical performance of artificially synthesized fibers, they are quite distinct from the true highly ordered hierarchical structure of natural spider silk (Teulé et al., 2007; Tucker et al., 2014). Even when full or nearly full sequences of genes are replicated, the quality of the fibers differ greatly from that of naturally spun spider silks. It can be concluded that there are biochemical (secretory) and physiological (assembly and spinning) functions, and the possible contribution of less prominent compounds and synergic effects, that we do not seem to fully understand. Science still has not decoded all the secrets of natural spider silk biosynthesis that spiders have used every day for millions of years. A better understanding of the key features of this process allows for the development of more promising biomimetic protocols that can then generate ideas with broad and important implications for more accessible and durable advanced material production.

### Prospects for Spider Silk Self-Assembly Throughout Biosynthesis

By deciphering the secrets of natural spider silk synthesis, science can gain a lot of inspiration for high-performance silk replicating materials. The absolute uniqueness of natural spider silk biosynthesis lies in its transformation of water-soluble spidroins into solid eco-friendly high-performance fibers at ambient temperature and moderate pressure (Andersson et al., 2016). There are numerous studies documenting how spiders adapt their fibers, from the amino acid sequence to the nano- and microscale organization employed during spinning, which allows for them to tune silk properties by adjusting the fibers to various environmental conditions (Saravanan, 2006; Sponner et al., 2007; Pechmann et al., 2010). This is accomplished by reorienting the peptide chains of liquid spinning dope (the liquid material from which silks are formed) through the structure of the spinning gland and the effect of the spidroin terminal domains (Andersson et al., 2013). The spinning glands regulate the concentration of numerous ions, mainly hydrogen ions, in the spinning dope to regulate pH (Dicko et al., 2004). As the dope passes through the narrowing spinning channel, it is acidified and exposed to a high degree of shear (Jin and Kaplan, 2003; Breslauer et al., 2009). During this process, the terminal domains of spidroin amino acid sequences act as molecular regulators to improve alignment via physiologically controlled homodimerization (Hagn et al., 2010; Eisoldt et al., 2012; Kronqvist et al., 2014). Due to their less hydrophilic nature and in contrast to repetitive domains, activated terminals retain their nature to initiate the assembly of spidroins via the formation of micelles (Jin and Kaplan, 2003; Rising et al., 2006; Gaines et al., 2010). During extrusion, the micelles form globule-like secondary structures that change from a helical and/or random coil to a predominantly β-sheet structure when exposed to shear force (Kenney et al., 2002; Dicko et al., 2004).

The self-assembly and rearrangement of silk peptide chains at the molecular level results in a high degree of β-sheet stacking in the fibers, accounting for their excellent mechanical properties. The strength of spider silk fibers can also be largely related to the presence of more mechanically stable β-sheets that possess a high density of hydrogen bonds due to the alanine-rich repeating domains of its amino acid sequence (this mechanism was described earlier). Moreover, it was found that, depending on the size of nanocrystals in dragline silk, the fiber exhibits varying ultimate strength and viscosity (Nova et al., 2010). Notably, the mechanical properties of spider silk depend not only on the composition of the amino acid sequences of spidroins or structural organization of the fiber, but also on the reeling speed of silk fibers during extrusion. Thus, faster silk spinning produces a stiffer fiber, while a decrease of the spinning rate leads to a fiber with higher elastic properties being formed (Vollrath et al., 2001; Perez-Rigueiro, 2005; Wu et al., 2009). This is also proven by the fact that spiders can greatly manipulate the material properties of their fibers by adjusting the size of the β-sheet nanocrystals through changing the reeling speed of the fiber (Du et al., 2006). Consequently, spider silk represents a comprehensive composite material. By altering its composition and organization, a spider can accomplish improved silk functionality and adapt spinning to changing environmental conditions.

Another example of the amazing natural adaptation of spider silk is the supercontraction of fibers when exposed to water in the form of steam or liquid (Liu et al., 2005, 2019; Blackledge et al., 2009). Consistent with this observation, the diameter of the fiber increases while the fiber compresses up to 50% of its original length (Guinea et al., 2003; Pérez-Rigueiro et al., 2003). Supercontraction is most pronounced in natural dragline silk fibers, as opposed to tubuliform silks (Vierra et al., 2011). This phenomenon can initiate a mechanism for creating tension in webs when they are loaded with dew or rain (Guinea et al., 2003). Additionally, during supercontraction, the stiffness of the fiber reduces due to the loss of molecular structural order along the fiber axis (Yang et al., 2000). Therefore, supercontraction provides an important mechanism for adapting the properties of fibrous materials.

Based on studies on the natural silk adaptation process, it is possible to provide a basis for potentially new bioprospective and biomimetic programs to fabricate highly adaptive fibrous materials (Agnarsson et al., 2010). Using these data, material scientists can learn how to better control the variability of flexible physicochemical properties of innovative smart and responsive materials.

## Fabrication of Spider Silk-Based Hybrid Materials

Spider silk is of practical interest because of its enormous functional potential. Understanding the connection between the morphology of the structural elements of spider silk proteins and their biochemical composition, physicochemical properties, and their supramolecular organization opens up opportunities for the production of new high-performance silk-based materials. The combination of nanostructures with biomaterials offers great prospects for constructing innovative functional devices for a wide range of applications. Therefore, the purpose of this section is to provide information on the possibilities and modern approaches to managing spider silk properties regarding possible ways to improve its functional properties. This section also aims to explain experimental observations and to lend scientific credence to the use of modeling approaches for the development of new biocompatible silk-based materials. To rationalize information on possible approaches to the modification of spider silk, namely combining techniques for manipulating natural or recombinant spider silk, this section was divided into sub-sections.

**Figure 3:**
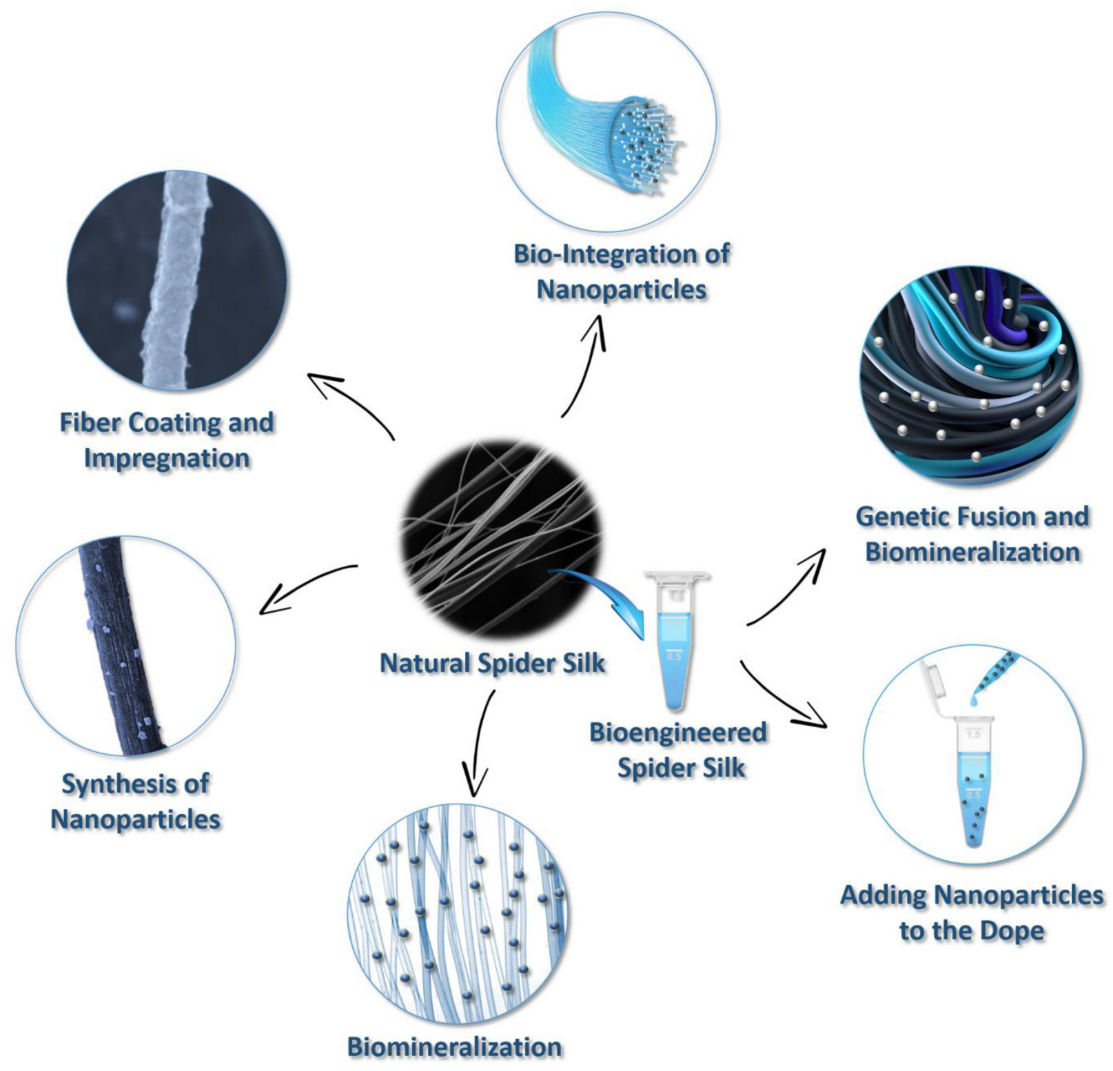
Summary of techniques for creating spider silk-based hybrid materials.

In spider silk, hierarchical protein assembly over several orders of magnitude is observed (Reches and Gazit, 2006; Knowles et al., 2010). The formation of hierarchical fibrous arrays is feasible via protein intra- and intermolecular interactions, suggesting that these micro-architectures can be used to integrate inorganic components within silk fibers to produce biopolymer hybrids with increased mineral loadings. Nevertheless, functional diversity can be achieved using the structural design of spider silk building blocks in combination with chemical approaches. Motivated by these innate natural nano-sized structures and designs, a series of fabrication strategies have been promoted to create hybrid organic-inorganic materials using spider silk proteins. These include biomineralization, impregnation, nanoparticle synthesis, bio-integration, and genetic fusions that are adopted to modify spider silks (Figure 3). These compositions have resulted in the development of functionally advanced hybrid materials for various applications.

Hybrid materials are widely used. For example, the improved biological and therapeutic properties of spider silk-based hybrid materials make it well-used in drug delivery, cancer-fighting therapies, as well as designing antimicrobial agents and osseous tissue replacement materials. The enhanced mechanical characteristics introduced by the inorganic phase generate prospects for using spider silk–inorganic particle-based hybrids as bone grafts or extra durable materials. Using optically active nanoparticles during the fabrication of functional spider silk-based hybrids provides more opportunities for their use in biomolecular detection, bioimaging, and in optoelectronic nanodevices. Noteworthy, spider silk supports electron transport between conductive nanoparticles in hybrid materials, which allows for the application of the resulting hybrids in sensing and energy spheres as bio- and vapor-sensors, energy harvesters, and microelectronic devices. Various methods of inorganic nanoparticles application to create said hybrid materials are discussed in the following sections in detail.

It is important to mention active studies on the strength characteristics and specific properties introduced by the doping phase of the obtained hybrid materials. The precentage of the doping inorganic phase in spider silk-based hybrids is often determined through differential scanning calorimetry (DSC) tests (Farokhi et al., 2014). Specific biological tests usually include cell adhesion and proliferation studies (Allmeling et al., 2008; Wohlrab et al., 2012) alongside the determination of antimicrobial ability (demarcating its inhibition zone by way of the agar diffusion method) (Wright and Goodacre, 2012). The mechanical properties of spider silk fibers, namely strength, extensibility, and toughness are extensively investigated using tensile tests (Giesa et al., 2011; Madurga et al., 2017). The impact of the structural organization within the silk fibers on their mechanical performance is also evaluated through their glass transition temperature (T_g_) (Guan et al., 2013, 2016). T_g_ is defined as the temperature range of glass transition, which is a reversible process occurring in material when its amorphous regions convert between glassy and rubbery states. The glass transition of silk-based materials directly correlates with the hydrogen-bond density and disordered fraction within silk (Hu et al., 2006). Thus, high T_g_ values typically display low extensibility yet high Young's modulus (comparison of the strength and stiffness) and breaking strength of the material (Wang Y. et al., 2014).

Atomic force (AFM) microscopy allows for surface structure, interactions and local mechanical properties to be investigated (Li et al., 1994; Kane et al., 2010; Brown et al., 2012; Menezes et al., 2013; Wang and Schniepp, 2018). The most straightforward charaterization methods for understanding the successful modification of silk fibers, however, are optical microscopy (Sponner et al., 2007; Zhao et al., 2017) and electron microscopy (Frische et al., 1998; Augsten et al., 2000; Du et al., 2006; Rousseau et al., 2007).

The relationship between the macroscopic properties and the microscopic structure of spider silks have been adressed in several works (Grubb and Jelinski, 1997; Glišović et al., 2008; Plaza et al., 2012), providing an explanation of the structure-function correlation of the material. For instance, to study the protein backbone solid ^13^C nuclear magnetic resonance (NMR) spectroscopy (Hronska et al., 2004; Holland et al., 2008; Wang et al., 2018b; Craig et al., 2019) and Raman spectroscopy (Sirichaisit et al., 2003; Colomban and Dinh, 2012) are frequently used. These methods are used to determine spidroin's secondary structure. Fourier-transform infrared (FTIR) spectroscopy and X-ray diffraction (XRD) analysis are two frequently used techniques when studying protein configuration and crystalline structure in silk-based materials. FTIR spectroscopy is a well-established experimental method used to study protein and polypeptide conformation, while synchrotron radiation FTIR (S-FTIR) microspectroscopy is succesfully applied in the investigation of silk spidroin conformation in single silk fibers (Ling et al., 2011). According to the method described by Madurga et al. (2017), FTIR spectroscopy provides information on spidroin secondary structure. The contribution of the secondary structures is attained from the mathematical processing of the amide I band (a characteristic band of polypeptide absorbance at about 1,650 cm^−1^). This method is extensively used for silk-based hybrid materials as it reveals the influence of the doping phases on the secondary sructure content in the protein backbone of the material. Both XRD and wide-angle XRD (WAXD) are also widely applied to study the crystalline structure, crystallite size and orientation in the materials (Sheu et al., 2004; Trancik et al., 2006; Sampath et al., 2012; Jenkins et al., 2013).

### Techniques for Creating Natural Spider Silk-Based Hybrid Materials

Hybrid materials based on spider silk proteins and metal nanoparticles for the immobilization of nanoobjects on biomolecules for use in biochemistry, biotechnology, and medicine are currently being extensively studied. Many proteins can be conjugated to metallic colloids by simply mixing them with a pre-synthesized metallic colloidal sol. The metal is usually bonded to the amines found along the protein backbone. Complementing at least two different types of materials causes the resulting hybrid material to display extraordinary properties. In this context, both natural and recombinant spider silk materials show outstanding mechanical and biocompatible properties. For high-performance fibers, natural spider silks are used to make composites with inorganic nanoparticles. Combination of the inorganic nanostructures and the biomaterials offers great opportunities in engineering innovative functional devices such as biosensors and actuators.

#### Fiber Coating and Impregnation Approaches

One of the most interesting approaches of spider silk coating was developed by Lee et al. (2009). In their work, spider dragline silk was used as a template for the deposition of zinc (Zn), titanium (Ti), and aluminum (Al) by multiple pulsed vapor-phase infiltration. Notably, the authors report the formation of metal-protein complexes with long exposure to water vapor resulting in the breaking of inner hydrogen bonds of the silk via bombardment from water vapor molecules. Thus, after long-term exposure to the metal precursor vapor, metal ions tend to bind to the broken bonding sites, resulting in the formation of metal-coordinated or covalent bonds. Due to this treatment, the toughness of hybrid spider silk fibers increases (Lee et al., 2009).

**Figure 4:**
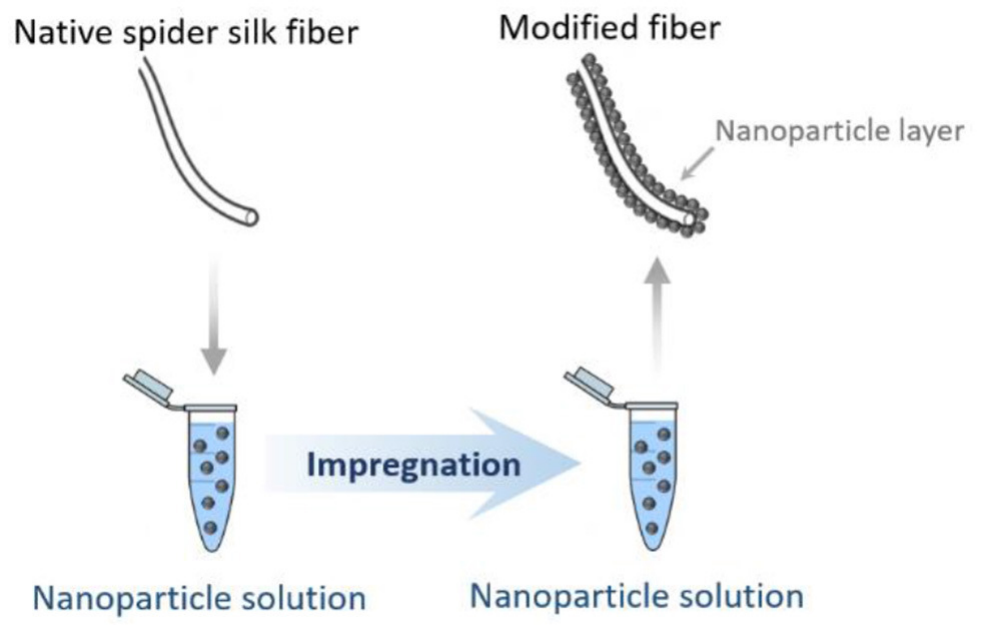
Schematic illustration of fiber coating and impregnation approach for spider silk-based hybrid material obtaining.

Another possible straightforward approach for obtaining natural spider silk fibers-based hybrid materials without significant changes to the protein backbone is by dip-coating natural spider silk fibers in nanoparticle suspensions. Natural spider silk–magnetite (Fe_3_O_4_) hybrids created via the aforementioned method have well-defined relatively stable coatings, possibly due to hydrogen bonding interactions at the oxide-silk interface. By exploiting the magnetic properties of these nanoparticles, such hybrid functional fibrous materials could be integrated into audio devices, where durable fabrics responding to a magnetic field are required (Mayes et al., 1998). The method is easy to perform and environmentally neutral, providing an opportunity for the routine fabrication of a variety of spider silk hybrids (Figure 4). As an example of the versatility of this approach, natural spider silk fibers dip-coated with hydrophobically functionalized gold nanoparticles were successfully fabricated in a similar way (Mayes et al., 1998). Additionally, a recent study on the fabrication of an optically active spider silk hybrid reports the successful attachment of ZrO_2_ and HfO_2_ upconversion nanoparticles to natural spider silk fibers by a simple and straightforward impregnation procedure. Namely, hybrids were formed by stirring of spider silk fibers in nanoparticle alcohol solution. It was shown that the upconversion luminescent properties of these nanoparticles could be extended to the macroscale components of the fibrous hybrid while the spidroin backbone remains unaffected. Such spider silk–inorganic nanoparticle-based hybrid materials may have prospective bio-applications in the fields of biosensing and bioimaging in a non-invasive and real-time manner (Kiseleva et al., 2019).

Another interesting approach to fabricating hybrid materials is the layer-by-layer electrostatic absorption method. For instance, spider silks were successfully coated with CdTe quantum dots by consecutively assembling CdTe nanocrystals and polyelectrolyte macromolecules onto spider silk fibers. The spider silk–CdTe hybrids exhibited core–shell structure characteristics and emitted extremely bright fluorescence while spider silk's mechanical properties were unaffected. This fluorescent spider silk may find applications in microelectronics and biomedicine (Chu and Sun, 2007).

Understanding the compatibility between spider silk and conducting materials is essential to further applications of spider silk in electronics. Therefore, noteworthy is the water-based and shear-assisted coating method of fabricating tough, versatile, flexible, and multi-functional spider silk–carbon nanotube hybrid fibers. Recent research has shown that the strong affinity of amine-functionalized multi-walled carbon nanotubes for spider silk is due to the structural changes in the carboxylic acid of spider silk. The charge carrier transport in those hybrids was primarily driven by inter-tube charge hopping. The conductivity of hybrid fibers was reversibly sensitive to strain and humidity, leading to custom-shapeable sensors and actuating devices (Steven et al., 2013). In the previously named study, the same group deposited a thin metallic film of gold nanoparticles to obtain sufficiently flexible natural spider silk–gold hybrid fibers to be used as electrodes in microelectronics (Steven et al., 2011). Additionally, multifunctional hybrid fibers with outstanding flexibility and conductivity were fabricated by wrapping a thin film of carbon nanotubes on natural spider silk. The fabrication of carbon nanotube-wrapped spider silk was done using the dry-coating and wet-collapsing method. The carbon nanotube film was adhered to the spider silk surface through ethanol action. The hybrid spider silk–carbon nanotube fiber could direct cell growth and simultaneously record signals evoked from cell beating without degradation over an extended period. Moreover, such a thin coating did not radically influence the mechanical properties of spider silks (Hou et al., 2018).

#### Nanoparticles Synthesis Using Natural Spider Silk

**Figure 5:**
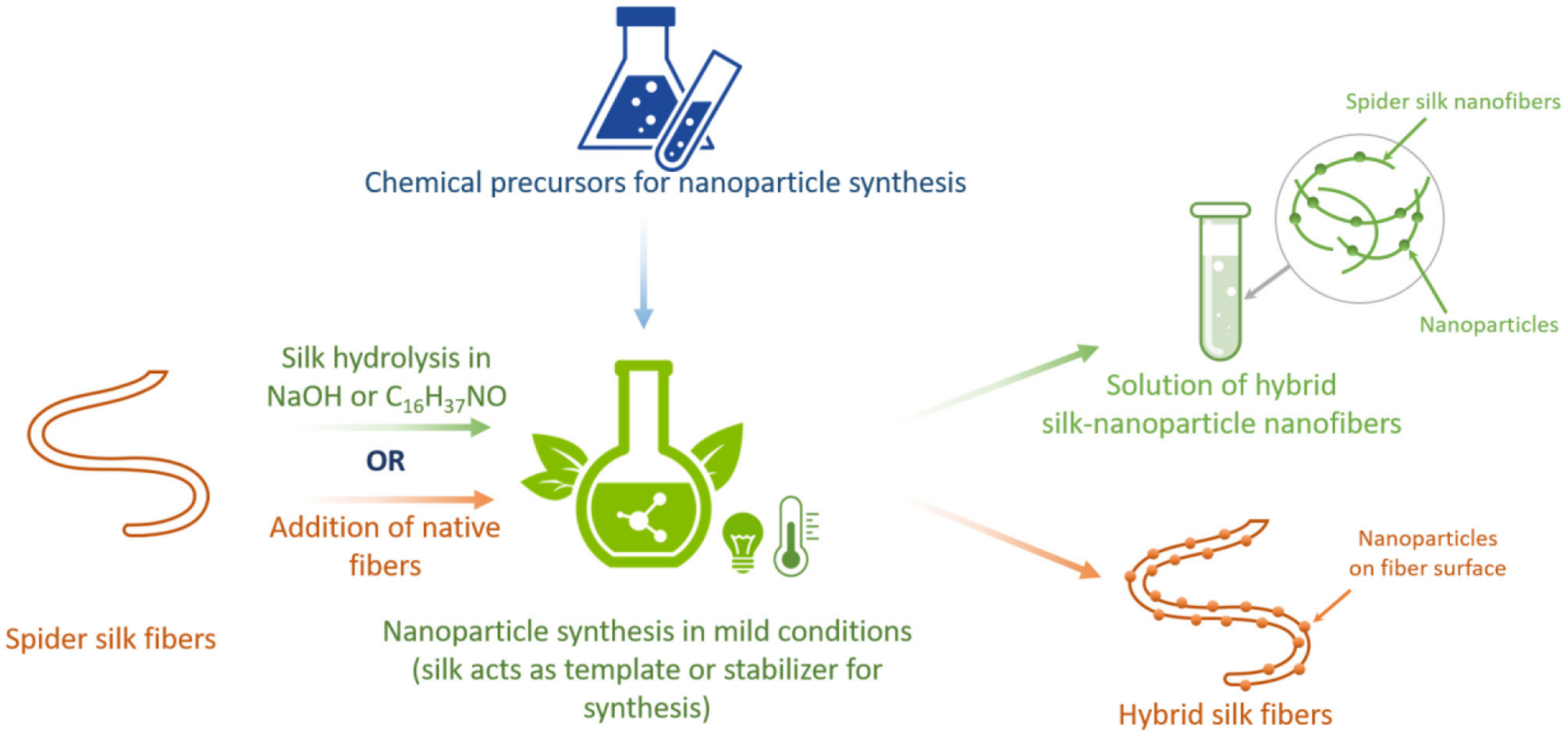
Schematic illustration of using natural spider silk for the synthesis of nanoparticles in hybrid material formation.

Spider silk has been used as a template in the synthesis of nanoparticles. This nanoparticle synthesis approach is of interest because of the simplicity of the process, its eco-friendliness, and the reduced amount of chemicals used (Salem et al., 2014). The utilization of biological substances and macromolecules during the synthesis of inorganic nanoparticles allows to avoid the production of unwanted or harmful by-products. Spider silk was recently reported to serve as an appropriate biological capping and stabilization agent that contributes to the steady rise of the synthesis of nanoparticles. In this technique, spider silk acts as an excellent scaffold for one-step nanoparticle synthesis (Figure 5). This approach results in the formation of environmentally friendly hybrids based on nanoparticles and spider silk. It has been reported that naturally spun spider silk fibers were used in the synthesis of gold nanoparticle bioconjugates via the spontaneous reduction of gold ions at spider silk (Singh et al., 2007). Here, spider silk served as a template for fiber incubation in aqueous chloroauric acid. Apparently, the binding of the gold nanoparticles to the spider silk was due to the strong interaction of spidroin amine groups with the gold surface (Selvakannan et al., 2003). This approach illustrates that spider silk modulates electron transport between nanoparticles on the hybrid surface, making it a promising candidate for the development of materials for vapor-sensing applications (Singh et al., 2007).

Hydrolyzed natural spider silk is also possible to be used for the synthesis of nanoparticles. In this context, spider silk was hydrolyzed in sodium hydroxide (NaOH) and added to silver nitrate (AgNO_3_) solution for the reduction of silver ions. Spidroins served as capping and stabilization molecules during the synthesis of silver nanoparticles as the carboxylate groups obtained from the alkaline degradation of spider silk served as a reducing agent in the generation of silver nanoparticles, while COO^−^ and NH^2+^ groups stabilized the silver nanoparticles and prevented their precipitation. The resulting hybrid solution exhibited antimicrobial activities against several multi-drug resistant clinical bacteria. This approach can be used to fabricate materials that protect against a microbial attack (Lateef et al., 2016). The same group of scientists later documented anticoagulant and thrombolytic properties of these hybrid materials (Lateef et al., 2017). Another example of regenerated (or in other words dissolved) natural spider silk application for the preparation of spider silk fiber hybrids is the synthesis of magnetite (Fe_3_O_4_) nanoparticles. Magnetite nanoparticles were successfully attached to spider silk hydrated in tetrabutylammonium hydroxide (C_16_H_37_NO). The authors note the preservation of the chemical amino acid block structures of the spidroins during synthesis. The materials generated were biocompatible and showed antibacterial properties, proving their potential therapeutic applications (Singh et al., 2015).

Recently, a carbon nanofiber synthesis method was also demonstrated via simple pyrolysis, using natural spider silk as a precursor. The resulting materials exhibited superior oxygen reduction reaction activity compared to that of carbon nanofibers prepared using metal-free carbon catalysts in alkaline conditions, thus providing opportunities for unconventional microbial energy harvesting (Zhou et al., 2016).

#### Biomineralization

**Figure 6:**
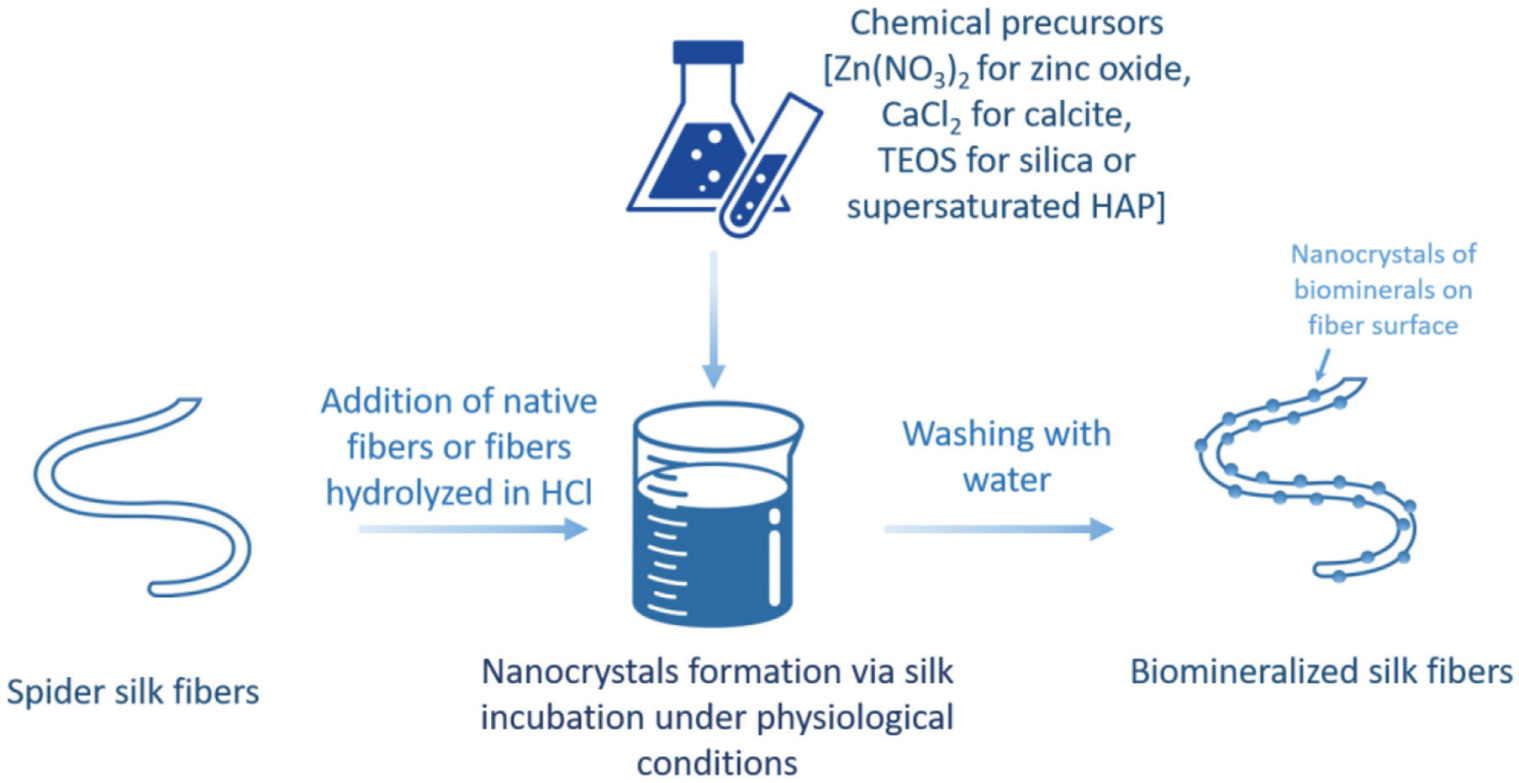
Schematic illustration of spider silk-based hybrid material production via biomineralization approach.

Recently, progress in biology has enabled the proteins and peptides responsible for the precipitation of inorganic materials within cells and controlling their nucleation and crystal growth (Figure 6) to be distinguished. This can be exploited for the mineralization of inorganic particles. The mechanism underlying this biomineralization approach of semiconductor metal oxides use hydrolyzed spider silk peptides, which include nucleophilic hydroxyls of cysteine, aspartic acid, and histidine amines. These specific groups bind, for example, zinc oxide (ZnO) nanoparticles and promote the crystal growth of hierarchical ZnO particles via the aggregation-driven mineralization with spidroin peptides under mild conditions. Such biomineralized ZnO materials coupled with spider silk may be used as biosensors for biomolecular detection or as optoelectronic nanodevices (Huang et al., 2008). Similarly, the biomineralization of natural spider silk fibers resulted in the crystallization of calcite on the spider silk substrate (Mehta and Hede, 2005). Likewise, spider silk–calcite hybrids were obtained with a pure calcite phase on the spider silk surface. The mechanical properties of spider silk complemented the inelastic ones of calcite, which is promising in the design of bone grafts for osseous tissue replacement materials (Dmitrović et al., 2016).

In the same way, it is possible to biomineralize spider silks with hydroxyapatite (HAP) via repetitive cycles of silk incubation in calcium phosphate rich solutions and washing with water. Notably, the biomineralization of natural spider silk fibers with hydroxyapatite yielded in the aligned orientation of c-axis of hydroxyapatite crystals along the spider silk fiber axis. This can be explained by the interactions between hydroxyapatite crystals and spidroins aligned along the long axis of the fiber by elongational flow during the natural silk spinning process. Moreover, hydroxyapatite crystal growth was consistent with the orientation of β-sheet crystals in the silk fibers (Cao and Mao, 2007). Both spider silk–calcium carbonate hybrids and spider silk–hydroxyapatite hybrids allowed for the production of new scaffold materials for bone tissue engineering or bone replacement materials (Mehta and Hede, 2005; Cao and Mao, 2007). In this context, a similar approach to creating spider silk–silica hybrids is to coat spider silk with silica precursors [such as tetraethylorthosilicate (TEOS)] and then subsequently heat it at 105°C. Later, the silk template can be removed via calcination at 600°C, resulting in the formation of materials with their pore structure determined by the silk template (Huang et al., 2003).

#### Bio-integration of Nanoparticles Into Natural Spider Silk

**Figure 7:**
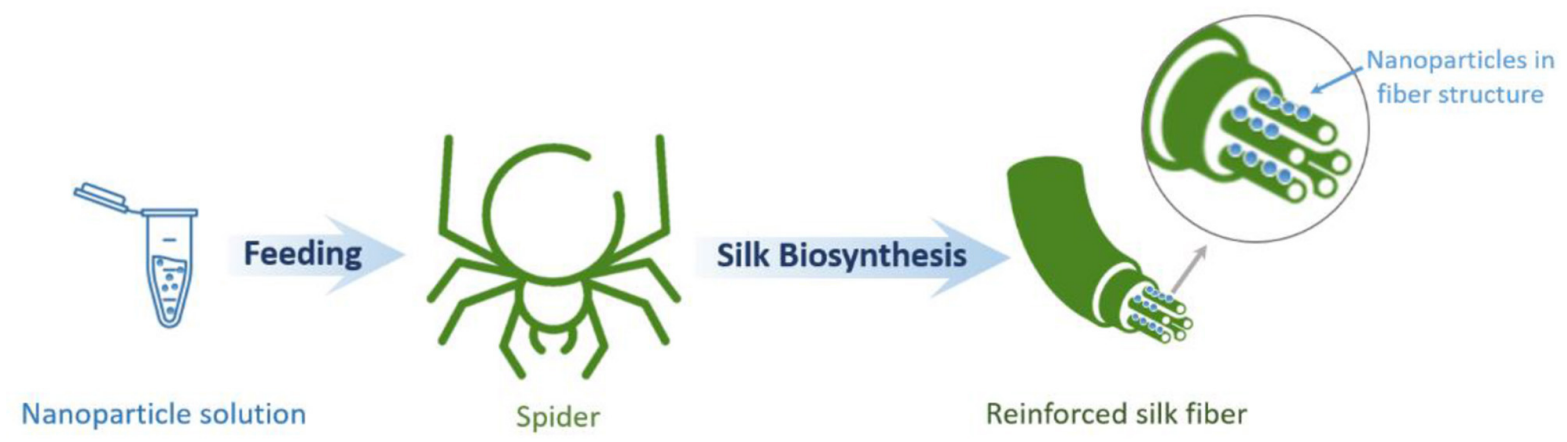
Schematic illustration of spider silk-based hybrid material formation through the bio-integration of nanoparticles into natural spider silk.

The evolution of biominerals in the protein matrix of natural materials is known to enhance their mechanical properties (Zhang, 2002). In this regard, it is possible to artificially incorporate diverse nanomaterials in spider silk protein structures aimed at improving the mechanical properties of the material. Thus, a method for producing spider silk fibers directly spun by spiders and reinforced by carbon nanotubes and graphene has been documented (Figure 7). Here, having been fed appropriate aqueous dispersions, spiders spun graphene and carbon nanotube incorporated silk. This approach yields fibers with greatly enhanced mechanical properties surpassing those of synthetic polymeric high-performance fibers. This observation indicates that the nanomaterials can be successfully inserted into the spider silk fibers. This approach of the natural integration of reinforcements in biological structural materials can be extended to other biological systems and lead to a new class of bionic hybrid materials (Lepore et al., 2017).

### Techniques for Creating Bioengineered Spider Silk-Based Hybrid Materials

Bioengineered spider silk can be functionalized to create hybrid materials with new functions or modified properties. Genetic modification of silk increases the ability of silk nanoparticles to bind and accumulate inside cells, which in turn improves the efficiency of drug delivery.

#### Hybrid Formation via Blending Nanoparticles With Spider Silk Proteins

Advanced antimicrobial hybrid materials were designed for sustainable drug release when combatting bacterial and fungal infections by combining the engineered eADF4(C16) spider silk protein and antimicrobial loaded silica nanoparticles. The silica–eADF4(C16) hybrids were made into different morphologies. The resulting hybrids showed excellent performance in terms of antimicrobial properties (Kumari et al., 2020).

**Figure 8:**
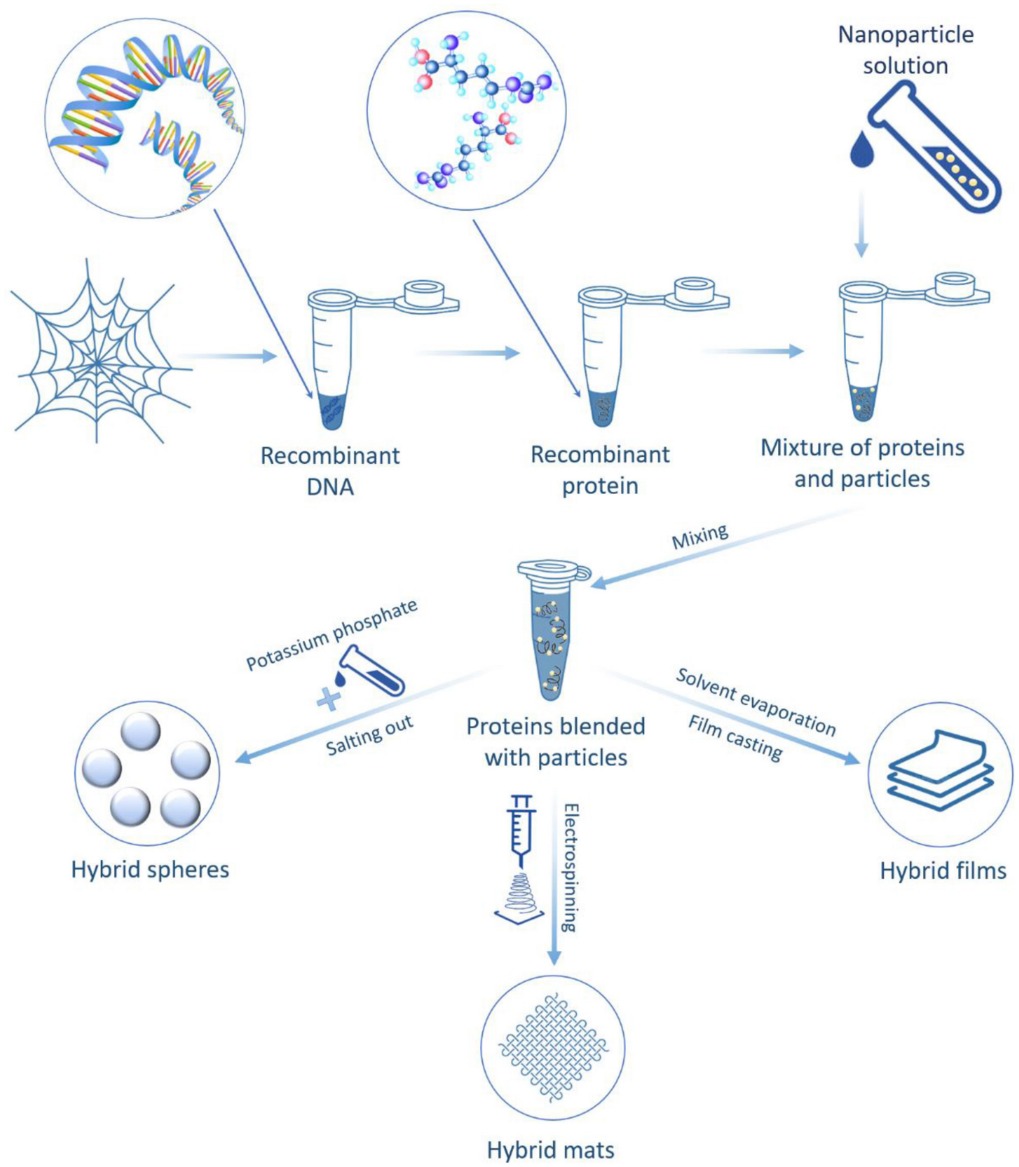
Schematic illustration of hybrid formation via blending nanoparticles with bioengineered spider silk proteins.

Kucharczyk et al. (2019) used three bioengineered spider silk proteins with different amino acid compositions derived from dragline silk proteins for hybrid production. These proteins were blended with iron oxide nanoparticle suspensions and formed spheres (Figure 8). The spider silk–iron oxide spheres were fabricated by salting the silk proteins–iron oxide nanoparticle suspension with a potassium phosphate solution. The resulting hybrid spheres showed promise to transport and release drugs. These nanomaterials have great potential in both magnetic resonance imaging applications and hyperthermia combined with drug delivery therapy against cancer cells (Kucharczyk et al., 2019). Another example is the fabrication of hybrid films made from recombinant spider silk proteins and single-walled carbon nanotubes. These materials exhibited exceptional mechanical properties due to stress transfer in the silk protein matrix to the inorganic filler and the potential for extensive matrix reorganization under applied stress (Blond et al., 2007).

Additionally, ceria (CeO) nanoparticles were added *in situ* to a recombinant spider silk solution and electrospun into hybrid nanofibers forming mats. In the hybrids, the embedded ceria nanoparticles introduced new mechanical and optical properties to spider silk nanofibers due to optical trivalent cerium ions, associated with the oxygen vacancies formed. Thus, the synthesized hybrids can be applied in different biomedical, sensing, energy spheres (Kandas et al., 2018).

#### Biomineralization and Genetic Fusion

Spider silk can assemble with organic and inorganic structures at different levels. It was shown that engineered spider silk binds with other structures via click chemistry and biotechnological approaches. In terms of biomineralization, the modification of recombinant spider silk proteins with specific binding motifs for hydroxyapatite (Huang et al., 2007), titanium dioxide, germania, and gold leads to various morphologies and allows for the control of organic-inorganic interfaces and structural features (Foo et al., 2006; Mieszawska et al., 2010a; Belton et al., 2012). The incubation of genetically engineered chimeric protein β-sheet rich films based on dragline spidroin and dentin matrix protein 1 in the simulated body fluid caused the growth of hydroxyapatite crystals on the film surface (Huang et al., 2007).

**Figure 9:**
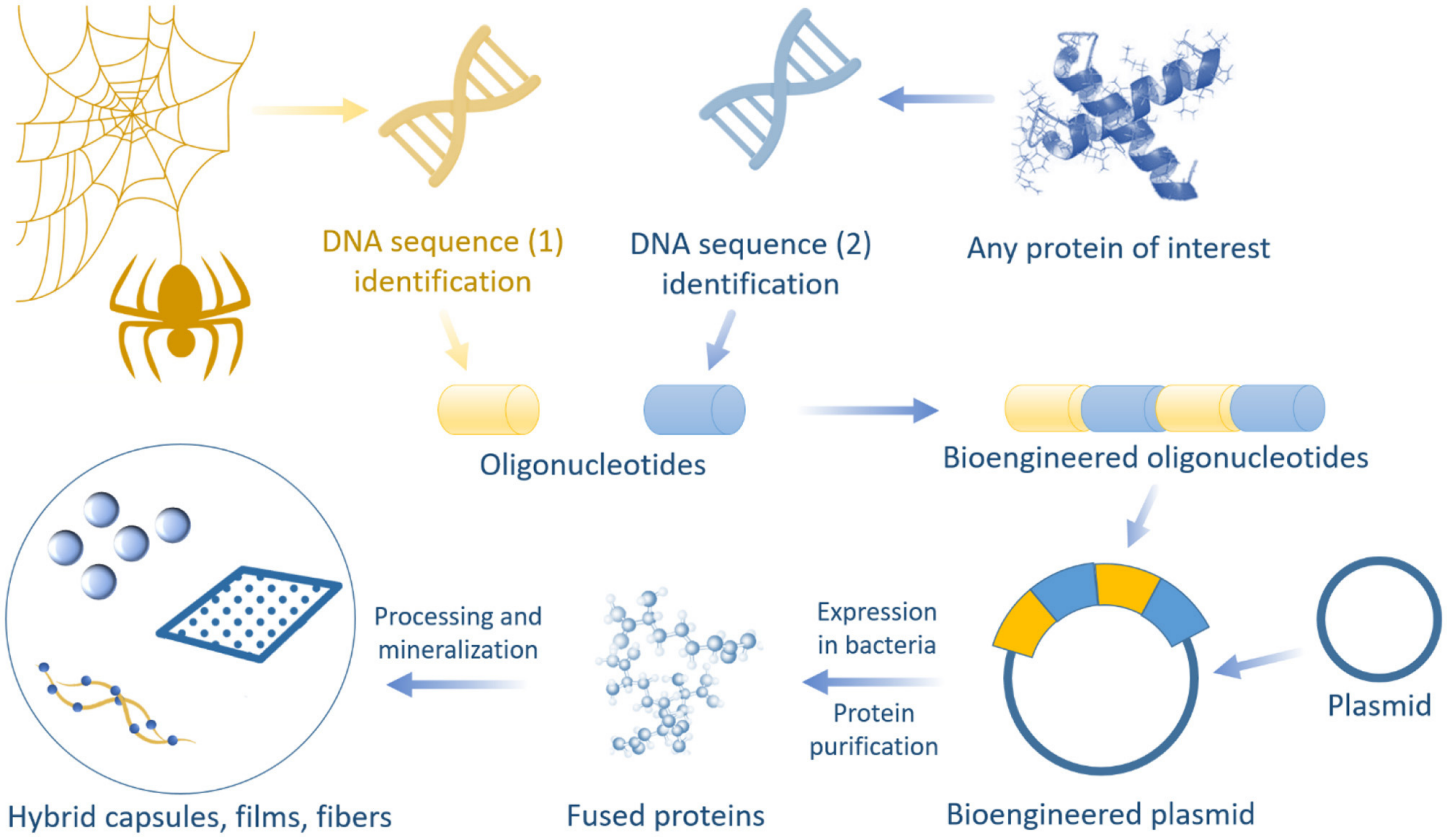
Schematic illustration of hybrid formation using genetic fusion and biomineralization approach.

Silaffin proteins (known as R5 peptide), responsible for silica mineralization in nature, are used for specific purposes. The genetically engineered chimeric spider silk proteins and R5 peptide have been reported to promote both self-assembly and biomineralization, as well as the biomimetic synthesis of spider silk–silica fusion proteins through combining the self-assembling domains of spider dragline silk and R5 peptides (Foo et al., 2006; Mieszawska et al., 2010b). With genetic control over nanodomain sizes and chemistry, as well as modification of synthetic conditions for silica formation, silk proteins self-assemble into highly stable β-sheet structures. The sizes and distributions of the silica components are controlled during the bioengineering process. The presence of silica in the silk films influenced osteogenic gene expression. These results indicate the potential use of these new silk–silica hybrid systems for bone regeneration. This approach can be extended to introduce alternative fusions of inorganic phases for other applications (Foo et al., 2006; Mieszawska et al., 2010b) (Figure 9).

An aqueous sol-gel process combined with microwave-assisted dissolution of hydrophobic synthetic spider silk was also shown to fabricate silk–silica hybrid particles. The incorporation of spider silk into the sol-gel process resulted in relatively spherical silk hybrids with tunable sizes and morphologies (Yang et al., 2010). The organo- and fluoro-silanes influence the spider silk secondary structures through varying β-structures in silk–silica hybrids. The ability to induce the defined secondary structures in the silk protein–silane hybrid particles may allow for bottom-up design of bioactive materials, where subsequent epitaxial growth and biomineralization can be tuned (Giasuddin and Britt, 2019). Additionally, thick homogeneous biomimetic crystalline calcium phosphate coatings were deposited onto the bioengineered spider silk fibers (produced from recombinant minispidroins) in a supersaturated simulated body fluid via the mineralization process. These hybrid fibers supported the attachment and growth of human mesenchymal stem cells, indicating prospective biomedical use of these functional materials (Yang et al., 2010).

Notably, two different biogenic materials, spider silk and magnetosomes, can be genetically combined, thereby generating a new hybrid composite with novel properties and enhanced application potential. In this respect, magnetosomes, which are natural magnetic nanoparticles with exceptional properties synthesized in magnetotactic bacteria via biomineralization, were used for encapsulation in biocompatible polymers to enhance their usability (Mickoleit et al., 2018). The genetic fusion of spider silk proteins with magnetosome membrane proteins were reported to enhance magnetite biomineralization and even cause the formation of a proteinaceous capsule, increasing the colloidal stability of the isolated particles. Furthermore, spider silk peptides fused to a magnetosome membrane protein can guide silk fibril growth on the magnetosome surface. This combination of two different biogenic materials generated a genetically encoded hybrid composite with new engineerable properties for various biotechnological and biomedical applications (Mickoleit et al., 2018).

## Conclusion

The range of spider silk applications is extremely broad due to its unsurpassed biophysicochemical properties and high degree of adaptability. Spider silk provides a good basis for the formation of hybrid functional materials with many uses—an option already being explored. However, it is clear that this field is only at its first stages of development based on the presented approaches for the synthesis of spider silk-based organic-inorganic hybrid materials. Moreover, large-scale industrial production of these materials is currently challenging due to some unavoidable difficulties related to natural spider silk fabrication. Commercially available artificial fibers are still far from natural as the structure and properties of natural spider silk are difficult for accurate reproduction. Furthermore, in comparison with more available silkworm silk, the nanostructure and macro-properties of spider silk vary significantly. The architecture and properties of natural spider silk fibers are still not fully researched. Therefore, further understanding of spider silk structure at both the molecular and supra-molecular levels, as well as its formation process is crucial for the development of more successful material modification and manipulation protocols. Based on these ideas, more accessible and durable advanced fibrous materials with tunable mechanical and biological properties can be generated. New evidence of hierarchical architecture within spider silk contributes to a better understanding of the possible integration of inorganic components within silk fibers to produce biopolymer hybrids with considerably improved functional properties.

## Author Contributions

AK, PK, and EK conceptualized the manuscript and completed the text. AK drafted the manuscript. All authors contributed to the article and approved the submitted version.

## Conflict of Interest

The authors declare that the research was conducted in the absence of any commercial or financial relationships that could be construed as a potential conflict of interest.
